# Body Composition as a Comorbidity-Independent Predictor of Survival following Nephroureterectomy for Urothelial Cancer of the Upper Urinary Tract

**DOI:** 10.3390/cancers15020450

**Published:** 2023-01-10

**Authors:** Christoph Pickl, Simon Engelmann, Florian Girtner, Miodrag Gužvić, Bas W. G. van Rhijn, Valerie Hartmann, Sonja Holbach, Sebastian Kälble, Maximilian Haas, Bernd Rosenhammer, Johannes Breyer, Maximilian Burger, Roman Mayr

**Affiliations:** 1 Department of Urology, St. Josef Medical Center, University of Regensburg, Landshuterstr. 65, 93053 Regensburg, Germany; 2 Department of Surgical Oncology (Urology), Netherlands Cancer Institute—Antoni van Leeuwenhoek Hospital, 1066 CX Amsterdam, The Netherlands

**Keywords:** sarcopenia, fat distribution, prognosis, skeletal muscle, urothelial neoplasm, transitional cell carcinoma

## Abstract

**Simple Summary:**

Urothelial carcinoma is a highly aggressive cancer. In addition to the further development and innovation of therapeutic strategies, research into risk and prognostic factors plays a major role. Comparatively little is known about the physical constitution of patients and its impact on the prognosis of the disease. The aim of this retrospective study was to assess specific parameters of the body composition of patients undergoing nephroureterectomy due to urothelial carcinoma of the upper urinary tract, in addition to the usual criteria with regard to commonly used ones. Computed tomography-based measurements were used to determine muscle mass and fat distribution. The cohort included 142 patients. We were able to demonstrate that loss of skeletal muscle mass is a significant comorbidity-independent risk factor. Visceral fat, on the other hand, seems to be protective. In conclusion, specific parameters of body composition can contribute to patient-specific risk stratification.

**Abstract:**

Radical nephroureterectomy (NUE) is the gold standard treatment for high-risk urothelial cancer of the upper urinary tract (UTUC). Besides sarcopenia and frailty, fat distribution is moving increasingly into focus. Components of body composition were assessed in patients undergoing NUE due to UTUC. The study cohort included 142 patients. By using CT-based measurements, the skeletal muscle index (SMI), subcutaneous adipose tissue index (SATI), and visceral adipose tissue index (VATI) were measured at the height of the third lumbar vertebra. Overall survival (OS) and cancer-specific survival (CSS) were estimated using univariable und multivariable Cox regression models. The prevalence of sarcopenia in the study population (*n* = 142) was 37%. OS and CSS were significantly reduced in sarcopenic patients. In the multivariable cox regression analysis, including age, ACE-27, T-stage, R-stage, LVI and necrosis, sarcopenia remained a significant risk factor of OS (HR, 1.77; 95% CI 1.02–3.07; *p* = 0.042) and CSS (HR, 2.17; 95% CI 1.18–3.99; *p* = 0.012). High visceral adipose tissue seems to be protective, although not statistically significant. Sarcopenia is a comorbidity-independent risk factor in patients who underwent NUE due to UTUC. Visceral fat represents a potentially protective factor. These results suggest that specific factors of body composition can be used for better risk stratification.

## 1. Introduction

Upper tract urothelial carcinoma (UTUC) accounts for roughly 5–10% of urothelial cancers, but its incidence is currently increasing. The reasons for this seem to be improved diagnostics and the resulting longer bladder cancer survival [1,2]. Radical nephroureterectomy (NUE) with bladder cuff excision is the gold standard for high-risk UTUC treatment [3]. Besides cancer related factors, many other prognostic risk factors, such as age, tobacco consumption, comorbidity indices, and surgical delay have already been identified [4]. In addition, sarcopenia, the loss of skeletal muscle mass and function, has been described as a risk factor for decreased overall survival (OS) after NUE [5,6]. Several studies have already shown that sarcopenia represents a risk factor concerning the survival of diverse tumor entities. Mayr et al. demonstrated that sarcopenia is an independent predictor for OS and cancer specific survival (CSS) for patients undergoing radical cystectomy for bladder cancer [7]. Besides sarcopenia and frailty, the complex body composition is moving increasingly into focus. For example, the distribution of fat (SATI, subcutaneous adipose tissue index; VATI, visceral adipose tissue index) has been investigated in gastric cancer patients. Cachexia patients with low SATI showed shorter survival than those with high SATI, while low VATI could not show a significant difference [8]. In this study, we aimed to investigate the influence of body composition in addition to sarcopenia in patients suffering from UTUC who underwent NUE.

## 2. Materials and Methods

### 2.1. Patients and Data Acquisition

After obtaining ethics approval (approval number: 18-965-101), 182 patients who underwent NUE with bladder cuff excision due to UTUC between 2004 and 2019 were selected. The patients were only included if histopathological results verified urothelial carcinoma. Patients without available staging computed tomography (CT) were excluded (*n* = 13). Eighteen patients were not accessible for follow-up. Patients who underwent radical cystectomy before NUE (*n* = 9) were excluded. In total, the study cohort included 142 patients. Demographic data, such as age, gender, and body mass index (BMI) are depicted in Table 1. Staging of UTUC was performed according to 8th edition of UICC TNM Classification. Among others, the date of follow-up, status of disease progression, and the cause of death were collected. Patients who suffered from metastatic disease at the time of death were assigned to “dead of disease”.

### 2.2. CT-Based Measurement of Body Composition

For measurements we used Osirix DICOM Viewer version 12. The Grow Region (2D/3D Segmentation) tool was used to select different tissue automatically. Intravenous contrast was not mandatory. The automatically created model was corrected manually.

1. Measurement of muscle tissue: As in various other sarcopenia studies, the cross-sectional skeletal muscle surface (cm^2^) was measured at the height of the third lumbar vertebra (L3) on two consecutive transversal computed tomography images. The range of –30 to +110 Hounsfield units was used for skeletal muscle [7,9].
2. Measurement of subcutaneous and visceral fat tissue: The measurement of fat distribution was also done at the L3 level on two consecutive transversal CT images. Adipose tissue was identified as Hounsfield units –150 to –50. Visceral adipose tissue (VAT) and subcutaneous adipose tissue (SAT) were normalized for height in metres squared, resulting in visceral adipose tissue index (VATI) and subcutaneous adipose tissue index (SATI) [10].

**Figure 1:**
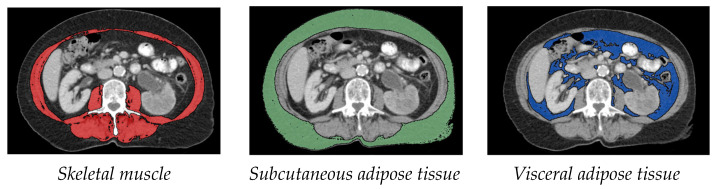
Axial computed tomography images at the third lumbar vertebra region. Ranges of Hounsfield units were used for quantifying different tissues.

Exemplary measurements are shown in Figure 1. The average of two measurements was used for the calculation.

### 2.3. Sarcopenia

Muscle area (cm^2^) was normalized for height in metres squared (m^2^) and reported as skeletal muscle index (SMI cm^2^/m^2^). We used the Martin criteria to define sarcopenia [11]:

Men BMI < 25 kg/m^2^:     SMI < 43 cm^2^/m^2^

Men BMI ≥ 25 kg/m^2^:     SMI < 53 cm^2^/m^2^

Women independent of BMI:   SMI < 41 cm^2^/m^2^

### 2.4. Comorbidity Survey

Comorbidities were quantified using the Adult Comorbidity Evaluation 27 (ACE-27) and American Society of Anesthesiology (ASA) score [12].

### 2.5. Statistical Analysis

All statistical analyses were performed using IBM SPSS Statistics Version 28.0 (IBM, Armonk, NY, USA). Continuous data are presented as median with interquartile range (IQR). After testing for normal distribution, differences between the sarcopenic and the non-sarcopenic collective were investigated using the Chi-square and Fisher`s exact tests. For ordinal or non-normally-distributed variables the Wilcoxon-Mann-Whitney U test was performed. A survival analysis was conducted using Kaplan-Meier and Cox regression analyses. Hazard ratios (HRs) and 95% confidence intervals (CIs) were calculated by univariable and multivariable Cox regression analyses. If significant predictors, defined by a *p*-value below 0.05, were found in univariable analyses, they became part of the multivariable analysis. For adipose tissue there are no internationally validated cut-offs. According to Labeur et al., the gender-related median was calculated and the cohort was divided into high- and low-VATI and high-and low-SATI-groups [13].

## 3. Results

**Figure 2:**
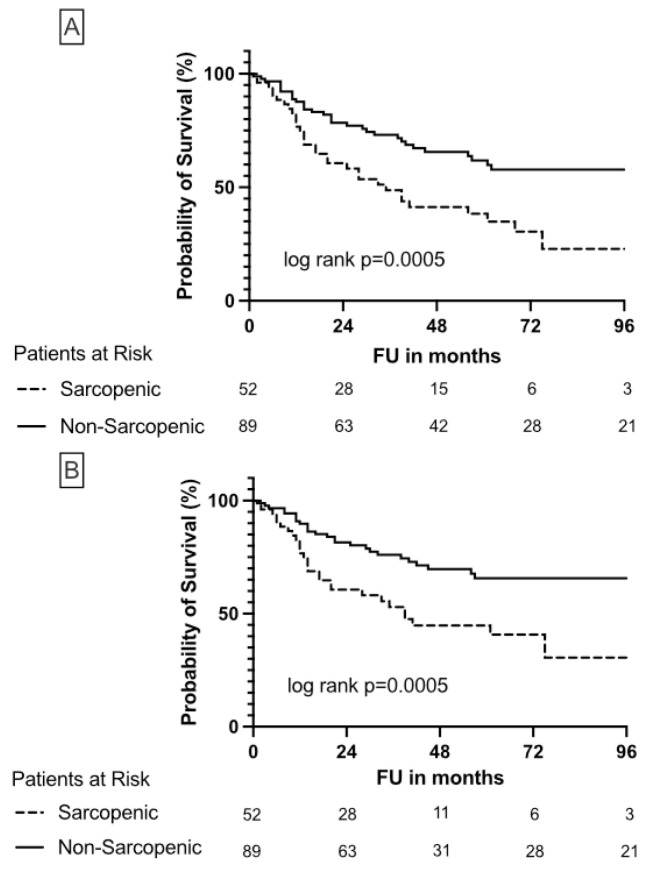
Kaplan-Meier plots illustrate overall survival (**A**) and cancer-specific survival (**B**) in sarcopenic and non-sarcopenic patients undergoing nephroureterectomy due to UTUC.

The patient characteristics are shown in Table 1. The prevalence of sarcopenia in the study population was 37%. In total, 60% of sarcopenic patients were male. In addition, sarcopenic patients were significantly older (median preoperative age 76 vs. 68 years; *p* = 0.001), and had a significantly lower BMI (24.9 vs. 26.7 kg/m^2^; *p* = 0.006) and more severe comorbidities. Specifically, 8/53 (15.1%) sarcopenic patients were classified in the severe group of ACE-27, while the non-sarcopenic collective included only 3 of 89 patients (3.4%) in the severe group. Concerning gender, ASA-Score, diabetes mellitus, kidney function, pT-stage, pN-stage, R-stage, concomitant CIS (carcinoma in situ), LVI (lymphovascular invasion), tumor necrosis, focality, location, SATI, and VATI, no significant differences were found between both groups. The median follow-up of the entire cohort was 37 months (IQR, 17–68 months). Sarcopenic patients had a significantly (*p* = 0.002) decreased follow-up (24 months) compared to non-sarcopenic patients (42 months). Out of 66 (47%) patients who died, 56 (39%) died as a consequence of cancer, whereas OSS and CSS were significantly decreased in sarcopenic patients (Figure 2).

**Figure 3:**
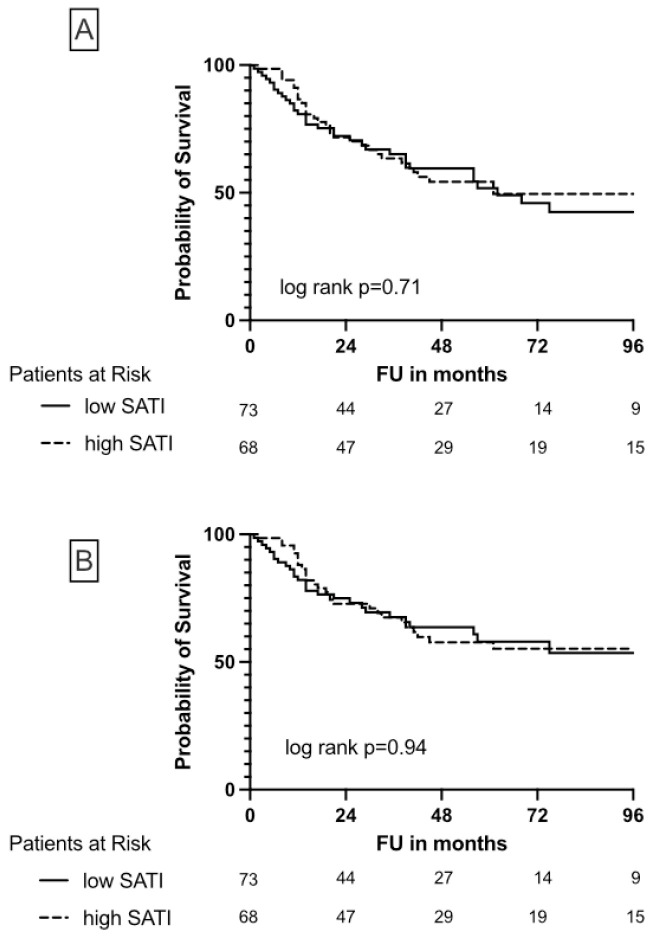
Kaplan-Meier plots illustrate overall survival (**A**) and cancer-specific survival (**B**) depending on high/low SATI in patients undergoing nephroureterectomy due to UTUC.

**Figure 4:**
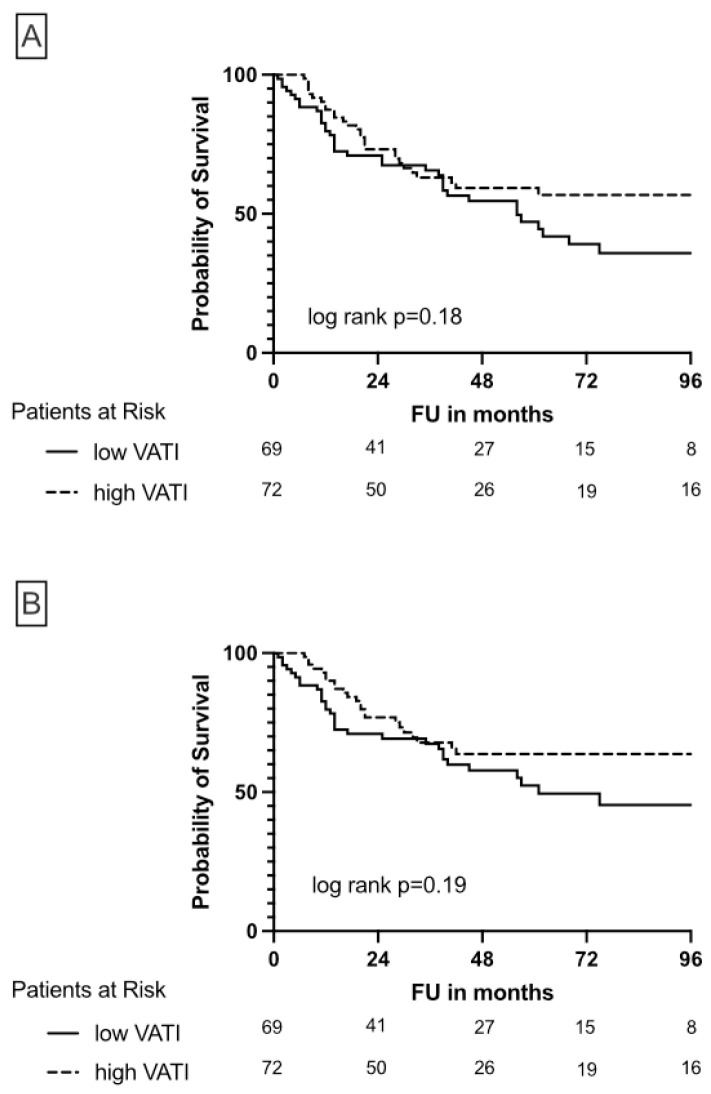
Kaplan-Meier plots illustrate overall survival (**A**) and cancer-specific survival (**B**) depending on high/low VATI in patients undergoing nephroureterectomy due to UTUC.

While SATI was not associated with differences in OS and CSS (Figure 3), high VATI seems to be protective in patients undergoing NUE due to UTUC, although this association was not significant (Figure 4).

The influence of diverse predictors of OS and CSS are listed in Table 2 and Table 3. In univariable cox regression models, age, ACE-27, sarcopenia, T-stage, R-stage, LVI and tumor necrosis were significantly associated with decreased OS (Table 2) and CSS (Table 3) after NUE due to UTUC. The N-stage was not considered in uni- and multivariable cox regression models because no systematic lymphadenectomy was performed (pNx 77.5%). Concerning BMI, only BMI <18.5 (“underweight”) represented a significant risk factor of cancer specific survival (Table 3). High VATI (continuous) showed a tendency towards statistical significance (*p* = 0.058) concerning CSS (Table 3). In the multivariable cox regression analysis, including age, ACE-27, T-stage, R-stage, LVI and tumor necrosis, sarcopenia remained a significant risk factor of OS (HR, 1.77; 95% CI 1.02–3.07; *p* = 0.042; Table 2) next to T-stage and LVI. Furthermore, in the multivariable cox regression analysis, including age, BMI, ACE-27, T-stage, R-stage, LVI and tumor necrosis, sarcopenia represented a significant risk factor of CSS (HR, 2.17; 95% CI 1.18–3.99; *p* = 0.012; Table 3) next to T-stage, BMI and LVI.

## 4. Discussion

There are already a number of studies in the literature dealing with sarcopenia in cancer patients. The objective of the present study was to assess the frequency of sarcopenia in patients suffering from UTUC [14]. Furthermore, we wanted to check whether it represents an independent risk factor. In this paper, we also wanted to study other factors of body composition. Special attention was paid to visceral and subcutaneous fat distribution.

One of the main findings of this study was that sarcopenia is a common phenomenon in patients suffering from UTUC. The prevalence of sarcopenia in our cohort was 37.3%. Studies of Fukushima et al. and Psutka et al. reported higher prevalences (58% and 68.8%, respectively), although they were determined with different cut-off values [5,15]. However, Psutka et al. used different sarcopenia criteria than those defined by Fearon et al. [15,16]. On the other hand, we reported similar prevalences (37.7%) for bladder cancer [7]. A meta-analysis examining sarcopenia in patients with solid tumors in general put the sarcopenia rate at 35.3% [17]. This study, like ours, was guided by the Martin criteria [11]. In summary, sarcopenia is a common phenomenon in patients suffering from urothelial carcinoma of the upper urinary tract. Nevertheless, the rate does not appear to be higher than in other tumor entities.

Furthermore, we found that sarcopenia is a comorbidity-independent predictor of OS and CSS following NUE due to UTUC. In the multivariable Cox regression analysis, sarcopenia remained, next to T-stage and LVI, a significant risk factor of OS. These results are in line with the findings of other research groups [5,18]. Surprisingly, diabetes mellitus, ASA-score, and preoperatively determined creatinine did not prove to be significant risk factors. Concerning BMI only, the group “underweight” represented a significant risk factor of CSS. This underlines the value of sarcopenia in the field of risk stratification. In our opinion, sarcopenia is a significantly underestimated risk factor in everyday clinical practice.

Finally, we established that body composition parameters, such as fat distribution, give more detailed information than BMI. Specifically, visceral fat represents a potentially protective factor in patients undergoing NUE due to UTUC. There are many studies that have looked at body composition, fat distribution, and its impact on cancer survival. In addition to sarcopenia, fat distribution seems to be becoming more prominent in risk stratification. Our study was able to demonstrate that high visceral adipose tissue (VATI) is associated with better outcomes after NUE due to UTUC, although they were not statistically significant. For subcutaneous adipose tissue (SATI), we could not see any statistically significant effect. Our findings are in line with studies that have looked at fat distribution as a potential risk factor in other cancer entities. For example, Matsui et al. could show that the loss of preoperative skeletal muscle mass and a decrease in visceral adipose tissue have an effect on the long-term outcomes of advanced gastric cancer patients. It has been described here that high visceral fat is associated with improved postoperative outcomes of patients with upper gastrointestinal cancer [19]. In addition, for unresectable advanced pancreatic cancer, a decline in visceral adipose tissue was linked with shorter survival [20]. In line with these findings, it has been shown that substances secreted by the adipose tissue have an impact on the biology of bladder cancer [21]. Martini et al. also identified a high visceral fat content as a protective factor in advanced urothelial carcinoma patients treated with immune checkpoint inhibitors [22]. On the other hand, Pan et al. did not find that fat parameters had any effect on the prognosis of UTUC patients. Here, a larger patient population was studied and different markers of the body composition (mean muscle attenuation, total abdominal muscle area, intermuscular fat tissue, total psoas muscle area, visceral and subcutaneous fat density, perirenal fat thickness) were assessed, but no systematic comorbidity index was assessed [23].

Our study has some limitations. Due to the retrospective study design, functional parameters, such as the grip strength test, bioelectrical impedance, nutritional status, and dietary habits could not be collected. Also, other factors such as smoking, neutrophil to lymphocyte ratio (NLR), or previous intravesical instillation therapies could not be assessed because no complete data were available in this regard. Furthermore, UTUC is a rare cancer, and many patients suffer from bladder cancer in addition to or during the course of the disease. This leads to a difficult definition of the patient cohort. We decided to exclude patients who underwent radical cystectomy before NUE. There is great heterogeneity in the literature with regard to the recording of body composition. Numerous factors, markers, indices, and measurement methods exist, which makes it difficult to compare the data. Prospective multicenter studies should be performed to develop clear standards and tools. This can contribute significantly to risk stratification in the future. In addition, exercise- and nutritional programs could find their way into clinical practice, and it needs to be clarified whether patients with an unfavorable body composition could profit from a kidney-sparing approach.

## 5. Conclusions

In summary, it can be noted that specific factors of body composition, such as sarcopenia and fat distribution, can be used for better risk stratification in patients who are undergoing NUE with bladder cuff excision due to UTUC. In our opinion, efforts should be made to include innovative body composition tools (for example bioelectrical impedance) in the risk stratification of these patients [24]. Subsequently, it must be examined whether certain patients at risk can benefit from appropriate countermeasures such as nutritional programs and physical activity [25].

## Figures and Tables

**Table 1:** Patient characteristics.

	Entire Cohort *N* = 142	Sarcopenic *N* = 53	Non-Sarcopenic *N* = 89	*p*-Value
Age (years), median (IQR)	71 (64–77)	76 (70–79.5)	68 (61–74.5)	<0.001 *
Gender (male), *n* (%)	87 (61.3)	32 (60.4)	55 (61.8)	0.867
BMI, median (IQR)	25.75 (23.5–29.4)	24.90 (22.2–27.4)	26.70 (24.2–30.3)	0.006 *
BMI-Category, *n* (%)				0.019 *
<18.5	2 (1.4)	1 (1.9)	1 (1.1)	
18.5–24.9	57 (40.1)	27 (50.9)	30 (30.7)	
25–29.9	52 (36.6)	18 (34.0)	34 (38.2)	
>30	31 (21.8)	7 (13.2)	24 (27.0)	
Cystectomy in the course, *n* (%)	11 (7.7)	4 (7.5)	7 (7.9)	0.945
ASA-Score, *n* (%)				0.054
1	13 (9.2)	4 (7.5)	9 (10.1)	
2	65 (45.8)	19 (35.8)	46 (51.7)	
3	59 (41.5)	28 (52.8)	31 (34.8)	
4	5 (3.5)	2 (3.8)	3 (3.4)	
5	-	-	-	
6	-	-	-	
ACE-27, *n* (%)				0.016 *
None	27 (19)	5 (9.4)	22 (24.7)	
Mild	59 (41.5)	23 (43.4)	36 (40.4)	
Moderate	45 (31.7)	17 (32.1)	28 (31.5)	
Severe	11 (7.7)	8 (15.1)	3 (3.4)	
Diabetes mellitus, *n* (%)	41 (28.9)	11 (20.8)	30 (33.7)	0.099
Creatinine preop. (mg/dL), median (IQR)	1.15 (0.94–1.50)	1.2 (0.98–1.54)	1.15 (0.91–1.45)	0.378
pathol. T-stage, *n* (%)				0.687
pTa	38 (26.8)	13 (24.5)	25 (28.1)	
pT1	19 (13.4)	7 (13.7)	12 (13.5)	
pT2	18 (12.7)	8 (15.1)	10 (11.2)	
pT3	59 (41.5)	21 (39.6)	38 (42.7)	
pT4	8 (5.6)	4 (7.5)	4 (4.5)	
pathol. T-stage-Category, *n* (%)				0.711
pTa, pT1	57 (40.1)	20 (37.7)	37 (41.6)	
pT2	18 (12.7)	8 (15.1)	10 (11.2)	
pT3	59 (41.5)	21 (39.6)	38 (42.7)	
pT4	8 (5.6)	4 (7.5)	4 (4.5)	
pathol. N-stage, *n* (%)				0.929
pN0	19 (13.4)	4 (7.5)	15 (16.9)	
pNx	110 (77.5)	40 (75.5)	70 (78.7)	
pN+	13 (9.2)	9 (17.0)	4 (4.5)	
R-stage, *n* (%)				0.429
R0	127 (89.4)	46 (86.8)	81 (91.0)	
R1	15 (10,6)	7 (13,2)	8 (9,0)	
Concomitant CIS, *n* (%)	14 (9.9)	3 (5.7)	11 (12.4)	0.195
LVI, *n* (%)	38 (26.8)	14 (26.4)	24 (27.0)	0.943
Tumor necrosis, *n* (%)	28 (19.7)	14 (26.4)	14 (15.7)	0.122
Multifocality, *n* (%)	24 (16.9)	8 (15.1)	16 (18.0)	0.657
Location, *n* (%)				0.652
Renal pelvis	99 (69.7)	37 (69.8)	62 (69.7)	
Upper third	7 (4.9)	4 (7.5)	3 (3.4)	
Middle third	16 (11.3)	6 (11.3)	10 (11.2)	
Distal third	20 (14.1)	6 (11.3)	14 (15.7)	
Follow-up (month), median (IQR)	37 (17–68)	24 (12–57)	42 (21–88)	0.002 *
Overall mortality, *n* (%)	66 (46.5)	34 (64.2)	32 (36.0)	0.001 *
Cancer specific mortality, *n* (%)	56 (39.4)	30 (56.6)	26 (29.2)	0.001 *
SATI, median (IQR)	60.98 (39.7–81.6)	54.26 (39–81)	62.95 (39.9–82)	0.398
VATI, median (IQR)	68.00 (36.2–94)	64.51 (36.1–68.6)	68.44 (36.9–102.1)	0.398

* = statistical significance, *n* = count of patients (percentage) IQR = interquartile range; Abbreviations: BMI = Body Mass Index (kg/m^2^), ASA = American Society of Anesthesiology, ACE-27 = Adult Co-Morbidity Evaluation (ACE-27), CIS = Carcinoma in situ, LVI = Lymphovascular Invasion, SATI = subcutaneous adipose tissue index (cm^2^/m^2^), VATI = visceral adipose tissue index (cm^2^/m^2^).

**Table 2:** Univariate and multivariate Cox regression models describing the impact of the studied predictors for overall survival after nephroureterectomy (*n* = 142).

	Univariate Analysis			Multivariate Analysis		
Variables	HR	95% CI	*p*-Value	HR	95% CI	*p*-Value
Age at NUE	1.051	1.02–1.08	<0.001 *	1.01	0.98–1.05	0.438
Gender (ref.: male)	0.928	0.56–1.53	0.772	-	-	-
Cystectomy in the course (ref.: absence)	0.776	0.31–1.93	0.586	-	-	-
BMI-Category (ref.: normal weight)						
<18.5 (underweight)	4.22	0.99–17.93	0.051	-	-	-
25–29.9 (Pre-Obesity)	0.71	0.40–1.23	0.219	-	-	-
>30 (Obesity)	0.73	0.38–1.38	0.329	-	-	-
ASA-Score (ref.: 1)						
2	1.29	0.45–3.69	0.635	-	-	-
3	1.81	0.64–5.13	0.262	-	-	-
4	2.90	0.64–13.09	0.165	-	-	-
5	-	-	-	-	-	-
6	-	-	-	-	-	-
ACE-27 (ref.: none)						
Mild	1.77	0.80–3.91	0.160	1.00	0.39–2.58	0.994
Moderate	2.29	1.03–5.09	0.041 *	2.12	0.85–5.29	0.108
Severe	3.54	1.28–9.81	0.015 *	2.40	0.76–7.61	0.136
Sarcopenia (ref.: absence)	2.34	1.47–3.89	<0.001 *	1.77	1.02–3.07	0.042 *
Diabetes mellitus (ref.: absence)	1.48	0.89–2.45	0.133	-	-	-
Creatinine preop. (continuous)	1.036	0.87–1.24	0.692	-	-	-
pathol. T-stage (ref.: pTa)						
pT1	0.76	0.20–2.86	0.685	-	-	-
pT2	2.07	0.75–5.70	0.162	-	-	-
pT3	4.60	2.15–9.85	<0.001 *	-	-	-
pT4	13.22	4.89–35.67	<0.001 *	-	-	-
pathol. T-stage-Category (ref.: pTa, pT1)						
pT2	2.24	0.87–5.79	0.095	1.41	0.50–4.02	0.516
pT3	5.00	2.56–9.76	<0.001 *	3.02	1.33–6.90	0.009 *
pT4	14.36	5.69–36.21	<0.001 *	6.28	1.90–20.72	0.003 *
R-stage (ref.: R0)						
R1	4.65	2.48–8.71	<0.001 *	1.96	0.85–4.51	0.116
Concomitant CIS (ref.: absence)	1.06	0.48–2.31	0.892	-	-	-
LVI (ref.: absence)	3.91	2.39–6.40	0.001 *	2.35	1.28–4.35	0.006 *
Tumor necrosis (ref. absence)	1.98	1.16–3.37	0.013 *	0.99	0.54–1.80	0.968
Multifocality (ref. unifocal)	1.31	0.72–2.41	0.382	-	-	-
Location (ref.: renal pelvis)						
Upper third	0.95	0.34–2.66	0.927	-	-	-
Middle third	0.76	0.33–1.79	0.533	-	-	-
Distal third	1.28	0.68–2.42	0.451	-	-	-
SATI (continuous)	0.98	0.99–1.01	0.431	-	-	-
VATI (continuous)	0.99	0.99–1.00	0.098	-	-	-

* = statistical significance, CI = confidence interval, HR = Hazard ratio, Abbreviations: NUE = Nephroureterectomy, BMI = Body Mass Index (kg/m^2^), ASA = American Society of Anesthesiology, ACE-27 = Adult Co-Morbidity Evaluation (ACE-27), CIS = Carcinoma in situ, LVI = Lymphovascular Invasion, SATI = subcutaneous adipose tissue index (cm^2^/m^2^), VATI = visceral adipose tissue index (cm^2^/m^2^). ref. = reference.

**Table 3:** Univariate and multivariate Cox regression models describing the impact of the studied predictors for cancer specific survival after nephroureterectomy (*n* = 142).

	Univariate Analysis			Multivariate Analysis		
Variables	HR	95% CI	*p*-Value	HR	95% CI	*p*-Value
Age at NUE	1.05	1.02–1.08	0.001 *	1.00	0.97–1.04	0.822
Gender (ref.: male)	0.97	0.57–1.67	0.920	-	-	-
Cystectomy in the course (ref.: absence)	0.73	0.26–2.02	0.55	-	-	-
BMI-Category (ref.: normal weight)						
<18.5 (underweight)	4.62	1.08–19.81	0.039 *	17.96	3.35–96.39	<0.001 *
25–29.9 (Pre-Obesity)	0.71	0.39–1.30	0.273	0.92	0.45–1.87	0.822
>30 (Obesity)	0.68	0.33–1.38	0.282	1.57	0.64–3.86	0.324
ASA-Score (ref.: 1)						
2	1.72	0.52–5.68	0.372	-	-	-
3	1.80	0.54–5.99	0.336	-	-	-
4	2.41	0.40–14.53	0.336	-	-	-
5	-	-	-	-	-	-
6	-	-	-	-	-	-
ACE-27 (ref.: none)						
Mild	1.84	0.79–4.28	0.155	0.79	0.27–2.33	0.668
Moderate	1.97	0.83–4.69	0.126	1.69	0.57–4.96	0.341
Severe	3.31	1.11–9.91	0.032 *	2.01	0.57–7.12	0.278
Sarcopenia (ref.: absence)	2.56	1.50–4.34	<0.001 *	2.17	1.18–3.99	0.012 *
Diabetes mellitus (ref.: absence)	1.00	0.55–1.81	1.0	-	-	-
Creatinine preop. (continuous)	1.06	0.83–1.25	0.88	-	-	-
pathol. T-stage (ref.: pTa)						
pT1	1.52	0.34–6.80	0.583	-	-	-
pT2	4.08	1.19–13.94	0.025 *	-	-	-
pT3	7.67	2.72–21.65	<0.001 *	-	-	-
pT4	24.75	7.37–83.13	<0.001 *	-	-	-
pathol. T-stage-Category (ref.: pTa, pT1)						
pT2	3.48	1.22–9.92	0.020 *	2.55	0.70–9.29	0.156
pT3	6.54	2.90–14.79	<0.001 *	4.04	1.30–12.56	0.016 *
pT4	21.11	7.56–59.00	<0.001 *	10.21	2.36–44.14	0.002 *
R-stage (ref.: R0)						
R1	5.35	2.82–10.14	<0.001 *	2.52	0.98–6.46	0.055
Concomitant CIS (ref.: absence)	1.28	0.58–2.83	0.539			
LVI (ref.: absence)	4.62	2.71–7.86	<0.001 *	2.91	1.43–5.90	0.003 *
Tumor necrosis (ref. absence)	2.31	1.31–4.04	0.004 *	1.56	0.72–2.57	0.347
Multifocality (ref. unifocal)	1.62	0.87–3.01	0.129	-	-	-
Location (ref.: renal pelvis)						
Upper third	1.18	0.42–3.33	0.749	-	-	-
Middle third	0.61	0.22–1.72	0.351	-	-	-
Distal third	1.54	0.80–2.96	0.195	-	-	-
SATI (continuous)	1.00	0.99–1.01	0.459	-	-	-
VATI (continuous)	0.99	0.99–1.00	0.058	-	-	-

* = statistical significance, CI = confidence interval, HR = Hazard ratio; Abbreviations: NUE = Nephroureterectomy, BMI = Body Mass Index (kg/m^2^), ASA = American Society of Anesthesiology, ACE-27 = Adult Co-Morbidity Evaluation (ACE-27), CIS = Carcinoma in situ, LVI = Lymphovascular Invasion, SATI = subcutaneous adipose tissue index (cm^2^/m^2^), VATI = visceral adipose tissue index (cm^2^/m^2^). Ref. = reference.

## Data Availability

The data can be shared up on request.
